# Marriage of Prof. Powell

**Published:** 1883-02-20

**Authors:** 


### *MARRIAGE OF PROFESSOR POWELL.*

Professor Thos. S. Powell, our Senior Editor, having long been a widower, and having found, according to Scripture, that "It is not good for man to be alone," has at length taken to himself a charming lady of the Old Dominion. The Doctor is known to be a great lover and defender of woman, and next to woman in his heart's affections is his native State, Old Virginia. True to himself and to his characteristics, he turns, in the time of his desolation, to his native State, and in the little city of Salem, noted for its charming women, its cultured and refined society, its beauty of situation and its magnificent mountain views, he selects—in the exercise of his superior knowledge of "What Constitutes a true Woman"—Mrs. Jennie Miller, a beautiful, intelligent and charming widow, and on the 27th December they were united in the holy bonds of matrimony; and thus a star from the Old Dominion is added to the constellations of Georgia.

